# Anticoagulants and the Propagation Phase of Thrombin Generation

**DOI:** 10.1371/journal.pone.0027852

**Published:** 2011-11-18

**Authors:** Thomas Orfeo, Matthew Gissel, Saulius Butenas, Anetta Undas, Kathleen E. Brummel-Ziedins, Kenneth G. Mann

**Affiliations:** 1 Department of Biochemistry, University of Vermont, Colchester, Vermont, United States of America; 2 Institute of Cardiology, Jagiellonian University School of Medicine, Krakow, Poland; 3 Johnson & Johnson, Pharmaceutical Research and Development, Raritan, New Jersey, United States of America; Emory University School of Medicine, United States of America

## Abstract

The view that clot time-based assays do not provide a sufficient assessment of an individual's hemostatic competence, especially in the context of anticoagulant therapy, has provoked a search for new metrics, with significant focus directed at techniques that define the propagation phase of thrombin generation. Here we use our deterministic mathematical model of tissue-factor initiated thrombin generation in combination with reconstructions using purified protein components to characterize how the interplay between anticoagulant mechanisms and variable composition of the coagulation proteome result in differential regulation of the propagation phase of thrombin generation. Thrombin parameters were extracted from computationally derived thrombin generation profiles generated using coagulation proteome factor data from warfarin-treated individuals (N = 54) and matching groups of control individuals (N = 37). A computational clot time prolongation value (cINR) was devised that correlated with their actual International Normalized Ratio (INR) values, with differences between individual INR and cINR values shown to derive from the insensitivity of the INR to tissue factor pathway inhibitor (TFPI). The analysis suggests that normal range variation in TFPI levels could be an important contributor to the failure of the INR to adequately reflect the anticoagulated state in some individuals. Warfarin-induced changes in thrombin propagation phase parameters were then compared to those induced by unfractionated heparin, fondaparinux, rivaroxaban, and a reversible thrombin inhibitor. Anticoagulants were assessed at concentrations yielding equivalent cINR values, with each anticoagulant evaluated using 32 unique coagulation proteome compositions. The analyses showed that no anticoagulant recapitulated all features of warfarin propagation phase dynamics; differences in propagation phase effects suggest that anticoagulants that selectively target fXa or thrombin may provoke fewer bleeding episodes. More generally, the study shows that computational modeling of the response of core elements of the coagulation proteome to a physiologically relevant tissue factor stimulus may improve the monitoring of a broad range of anticoagulants.

## Introduction

The management of anticoagulant therapy has relied on clot-based assays such as the prothrombin time (PT) assay. In the case of warfarin therapy, it has been established in clinical studies that a prolonged clot time in the PT assay, after normalization to account for reagent variability (expressed as the International Normalized Ratio, INR) in the 2 to 3-fold range indicates a sufficient level of anticoagulation in many patients [1]. However, the efficacies of newer generation anticoagulants like dabigatran etexilate [2] and rivaroxaban [3] are not well represented by the PT assay. A generally applicable method to evaluate all classes of anticoagulants is lacking.

A limitation of clot based assays is that more than 95% of thrombin generation occurs after clot formation, whether studied in plasma [4] or whole blood [5] or in reconstructions of the coagulation proteome using purified proteins [6]. Post-clot thrombin generation (*i.e.* propagation phase) is often characterized in terms of parameters describing features of its dynamics, *e.g.* maximum (max) rates and levels of formation. Numerous studies have concluded that appropriate levels of propagation phase thrombin formation appear critical to the coagulation process via stabilization and maintenance of the barrier function of the blood clot [7]–[9]. In addition, methods that present the entire course of thrombin generation during a coagulation event demonstrate an increased capacity, relative to clot based assays, to distinguish between control individuals or between individuals characterized by the same inherited disorder of the coagulation process [10]. One source of this increased discrimination derives from the collective effects of normal range variation in coagulation factor or inhibitor concentrations between individuals combined with the use of a concentration of tissue factor (Tf) stimulus more consistent with that characterizing intravascular lesions [11] (*i.e.* low pM Tf versus 5 to 10 nM Tf in the PT assay).

Empirical evaluations of various anticoagulants using comprehensive thrombin generation assays have demonstrated dose-related effects on clot times and on post-clot thrombin parameters [12], [13] and have shown differences between anticoagulants with respect to the pattern of post-clot thrombin generation suppression. These studies however do not explicitly address the mechanistic basis for these differences between anticoagulants or differences in the response to a given anticoagulant among individuals that have been observed [14].

In this study, computational and empirical approaches are taken to relate propagation phase thrombin generation and anticoagulant efficacy. Specifically, analyses of thrombin generation during warfarin anticoagulation serve as a point of reference for empirical and computational studies detailing the dynamics of the suppression of thrombin generation for each of three anticoagulants currently in use or in clinical trials (unfractionated heparin (UFH), fondaparinux (Fpx), the direct fXa/prothrombinase (fXa-fVa) inhibitor rivaroxaban), and a generic reversible thrombin inhibitor.

## Results

### Model Representations of Warfarin Anticoagulated Individuals

We have previously shown that contact pathway inhibited blood samples from individuals with similar levels of warfarin anticoagulation (INR 1.9–2.5) and no reported bleeding pathology exhibit significant variability in their Tf-initiated coagulation response [15]. Plasma composition data determined at the time of that study for each of the warfarin treated and control individuals are used here to generate a model representation of the changes in Tf-initiated thrombin generation associated with warfarin anticoagulation. Table 1 (study 1) presents the mean factor composition values for an anticoagulated group (N = 7), one individual assessed 8 times over 6 months and the control group (N = 5) for this study. Table 1 (study 2) presents the mean factor composition levels from a separate study for a larger warfarin treated group (N = 47, shown as INR ranges of 2 to 2.4 and 2.5 to 2.9) and a matching control group (N = 32). There are no corresponding whole blood data for this second group. Levels of antithrombin, TFPI, fV and fVIII are within the normal range for all individuals in the two warfarin study groups, as are the VKD protein levels in the 2 control groups.

**Table 1:** Coagulation factor levels*.

	N	FII	FVII	FIX	FX	AT	FV	FVIII	TFPI
Study 1									
Control	5	98.0 (16.0)	83.4 (14.4)	107.8 (25.8)	86.4 (13.9)	94.6 (10.9)	101.6 (26.7)	149.4 (37.4)	91.7(4.4)
Warfarin (Group)	7	24.7 (5.3)	41.6 (11.8)	45.0 (12.7)	15.9 (4.7)	98.4 (8.9)	97.4 (27.0)	198.3 (47.3)	75.3(6.0)
Warfarin (Individual) †	8	24.9 (3.8)	34.8 (8.6)	37.9 (5.5)	15.0 (3.4)	107.0 (9.5)	138.9 (10.3)	126.6 (11.2)	86.0(11.6)
Study 2									
Control	32	110.7 (14.8)	107.7 (17.1)	120.2 (19.9)	119.1 (20.6)	100.0 (13.5)	108.7 (12.3)	139.5 (26.7)	114.3 (16.8)
Warfarin(INR 2.0–2.4)	25	35.4 (9.9)	30.5 (8.9)	32.3 (6.8)	37.2 (11.3)	108.0 (15.9)	104.3 (15.0)	132.3 (33.1)	104.2 (27.8)
Warfarin(INR 2.5–2.9)	22	26.2 (5.1)	31.9 (9.7)	34.4 (7.6)	37.7 (9.0)	103.7 (9.7)	106.8 (14.0)	117.2 (30.4)	110.3 (37.2)

*Mean (SD) expressed as a percentage of each factors mean physiologic level.

† One individual studied at eight separate times over a six month period.

**Figure 1:**
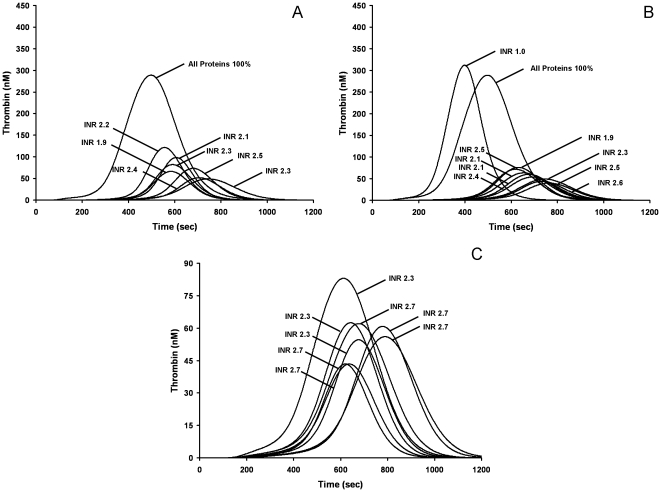
Model representation of individuals stablely anticoagulated with warfarin. Computationally derived thrombin generation profiles reflecting each individual's actual ensemble of factor levels are presented with each anticoagulated individual's profile identified by his clinically determined INR value. A thrombin generation profile characterizing a hypothetical individual with all factors at mean physiologic levels is also shown. Panel A, 7 individuals; Panel B, One warfarin treated individual assessed 8 times over 6 months and after warfarin therapy was ended (INR = 1); Panel C, 7 individuals from study 2 with INR values of 2.3 or 2.7. Note the difference in Y-axis scale.

Figure 1A presents cTGPs for 7 individuals from study 1 with INR values between 1.9 and 2.5. Figure 1B presents cTGPs for one warfarin anticoagulated individual from study one sampled over a 6 month period along with a cTGP representing that individual after the cessation of therapy (INR = 1). Direct inspection of the cTGPs in both Figures 1A and 1B shows that the relative intensity of thrombin generation is not explicitly related to the INR, consistent with the results of the whole blood study examining the response these individuals to a Tf stimulus of the same magnitude [15]. For example the cTGPs of two individuals each with INR values of 2.3 shown in Figure 1A are characterized by thrombin parameters max rate and max level that differ by 2 fold. Disparities of similar magnitude between the cTGPs of warfarin-treated individuals with the same INR are also observed in the larger warfarin groups. Figure 1C shows cTGPs from three individuals with INR 2.3 with those from 5 individuals with INRs of 2.7.

To develop general criteria to define a model representation of successful warfarin anticoagulation, thrombin parameters were extracted from the cTGPs of the individuals in each of the two empirically studied warfarin-treated groups, as well as the control group of that empirical study (5 control individuals or 32 individuals). The values of the thrombin parameters of the warfarin-treated groups were then expressed relative to the corresponding parameter values of the matching control group values (Table 2). The comparison of model predicted clot times between control and anticoagulated individuals yielded a ratio value analogous to the ratio (INR) between a patient's prothrombin time and that of a standard plasma sample. For the group of seven warfarin treated individuals in study 1, the average cINR (2.3±0.4) was consistent with the clinically determined INR values (2.2±0.2) and overlapped with the prolongation of clotting reported in the whole blood study of these individuals (2.1±0.6) [15]. Good correspondence was also observed when cINR and INR values for the one individual studied multiple times. Additional features of the cTGPs of these warfarin-treated individuals include a ∼70 to 80% suppression in the both the max rate and level of thrombin produced as well as total thrombin generated. Similarly, when the larger warfarin-treated group (N = 47, subdivided into two groups based on INR) was subjected to the same type of analysis and their cTGPs expressed as a ratio relative to their control group (N = 32), cINR values showed good correspondence with their respective plasma based INR values (Table 2).

**Table 2:** Computational analysis on individuals on warfarin.

	N	Fold Prolongation	% Max Rate	% Max Level	% AUC	Plasma INR
**Study 1** *						
Warfarin Group	7	2.3 (0.4)	29 (14)	24 (9)	20 (7)	2.2 (0.2)
Warfarin Individual†	8	2.5 (0.4)	17 (6)	17 (6)	18 (5)	2.3 (0.2)
**Study 2** ‡						
INR 2.0–2.4	25	2.38 (0.60)	18.2 (8.5)	20.4 (7.1)	25.2 (9.6)	2.2 (0.14)
INR 2.5–2.9	22	2.66 (0.70)	14.2 (8.5)	15.7 (6.2)	19.3 (7.0)	2.7 (0.14)

*Plasma composition data from individuals on warfarin and a control group (n = 5) were used to construct computational thrombin generation profiles from which thrombin parameters clot time, maximum rate of thrombin generation (Max Rate), maximum level of thrombin generation (Max Level) and total thrombin (AUC) were extracted. The parameter data are presented as mean (SD) for either a ratio or a percentage between warfarin treated and control individuals.

† One individual studied at eight separate times over a six month period.

‡ 47 anticoagulated patients were divided into two groups based on their INR and their thrombin generation modeled. Thrombin parameters were extracted and compared to the control group (n = 32) as in study 1.

We have previously reported synthetic coagulation proteome data comparing thrombin generation by an “average” warfarin-treated individual, constructed using the mean factor levels for the seven warfarin-treated individuals modeled here in study 1, with an “average” control individual, reflecting the mean physiologic values of the study 1 control group [16]. Consistent with the current computational analysis, the prior empirical reconstruction of the “average” warfarin treated individual displayed a 2.4 fold prolongation of the initiation phase (clot time) with a ∼85% suppression of the max rate and level of thrombin generation relative to the “average” control.

### Analyses of cINR and INR differences on an individual basis

**Figure 2:**
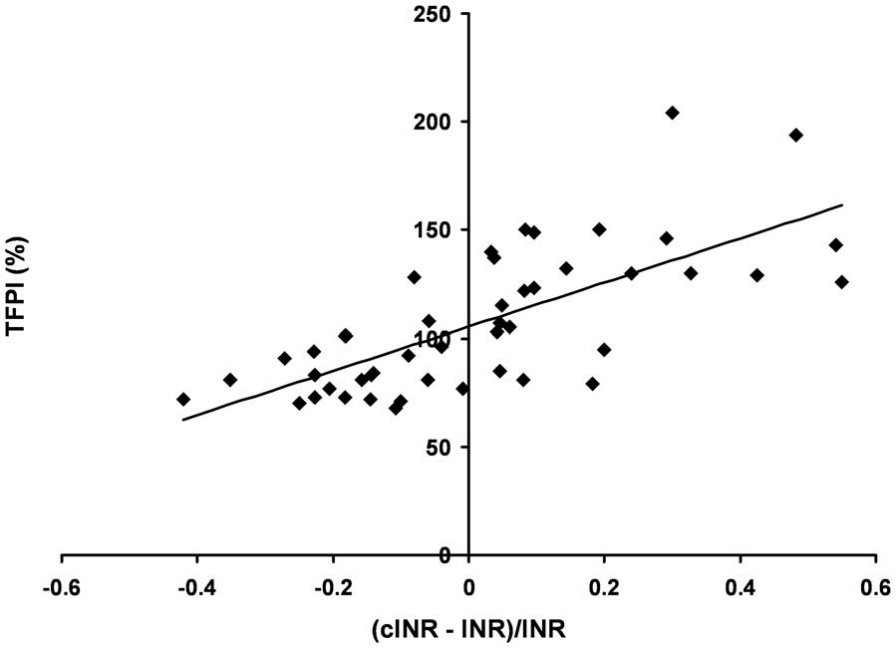
Dependence of differences between INR and cINR values on TFPI levels. The difference between each individuals (N = 47) INR and cINR was expressed as a percent of the INR [(cINR-INR)/INR] and then plotted against each individual's TFPI level expressed as a percent of mean physiologic. Individuals with high normal TFPI levels display positive ratio values while those with low normal values display negative ratios. Linear regression analysis yielded the following expression y = 101.65x + 105.4 with an r^2^ = 0.51. The predicted y intercept, representing the TFPI concentration where cINR and INR values do not diverge, correlates well with the mean TFPI value for the population (see Table 1). The three individuals that had a thrombotic event subsequent to the blood draw for compositional analysis are indicated in red.

When the correspondence between plasma derived INR values and cINR values was examined for each individual in study 2 (N = 47), we observed that in 19 (40%) cases the difference was less than 10%, but that more significant differences occurred in the majority of comparisons. We investigated the potential for these differences to derive from variation in specific factor levels by establishing a metric which quantified the relative disparity between model and PT assay derived values for each individual. Figure 2 presents the results of linking this metric for each individual to that individual's level of TFPI, as percent of the mean physiologic value, and then assessing the overall trend between the magnitude of the difference between the cINR and INR and TFPI level for the entire population. The result is a strong correlation between the magnitude and direction of the discrepancy and the TFPI level, as reflected in the r^2^ value (0.511, p<0.001). Similar analyses were formed with all other factors and yielded the following r^2^ values: fVII (0.217, p = 0.001), fVIII (0.199, p = 0.002), AT (0.122, p = 0.016), and for fII, fV, fIX and fX the r^2^ values were below 0.05 (all p>0.3). In contrast, for the same population, when plasma derived INR values for each individual were plotted directly against the levels of each of their factors, the correlation with fII was strongest (r^2^ = 0.37, p<0.001) followed by FVIII (r^2^ = 0.068, p = 0.08), with all others r^2^ below 0.02, including fVII (all p>0.3). Thus the computational analysis, which, unlike the PT assay, reflects the inhibitory effects of TFPI on thrombin generation, suggests that the effectiveness of the INR as a measure of an individual's level of anticoagulation may be lessened in individuals characterized by TFPI levels approaching the extreme of its normal range (46–171% of mean physiologic).

In the study 2 population, 11 of the 47 warfarin-treated individuals had cINR values below 2, suggesting they were potentially under anticoagulated. Three of these individuals suffered thrombotic events subsequent to their reported INR determination (individuals are indicated in Figure 2). INR values for these three were 2.1, 2.2 and 2.5 while their cINR values were 1.7, 1.8 and 1.5 respectively; respective TFPI concentrations were 77%, 73% and 72%.

### Empirical validation of modeling of coagulation proteome variation

Previously we have shown in both empirical [17] and computational approaches [18] that normal individuals exhibit significant phenotypic individuality in their Tf-dependent coagulation responses. In this study, when the 37 control individuals are considered collectively, modeling derived thrombin parameters such as max rate, max level and total thrombin vary over a 2 to 3 fold range in this group while predicted clot times range between ∼170 and 250 s, all well within the range reported when cTGPs from 473 control individuals from the Leiden Thrombophilia Study were analyzed [18].

**Figure 3:**
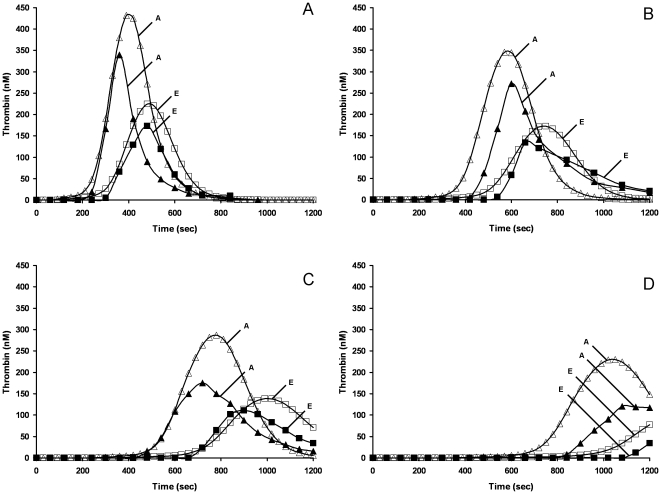
Empirical and computational studies of coagulation factor-based variability in thrombin generation in 2 control individuals in the presence or absence of rivaroxaban. cTGPs for 2 individuals (A,E) (open symbols) based on their plasma factor composition with corresponding empirical thrombin generation (closed symbols) measured in synthetic coagulation proteome reconstructions. Panel A, no rivaroxaban; Panel B, 4 nM rivaroxaban; Panel C, 10 nM rivaroxaban; Panel D, 21 nM rivaroxaban. Predicted thrombin concentrations are given at time points to match empirical sampling, which is performed at 1 minute intervals.

Figure 3A shows cTGPs for 2 control individuals (study 1), where every factor level of these individuals is within the normal range (Table 1). Thrombin parameters max rate, max level, and AUC differ ∼2 fold while the clot time parameter increased from 173 s (A) to 250 s (E). The differences between the two profiles derive from differences in plasma factor composition: in this case, all procoagulant factors (fII, fV, fVII, fVIII, fIX, fX) are at higher levels (33–62%) in individual A while TFPI and AT levels are similar between A and E. Also shown (Figure 3A) are two TGPs for synthetic proteome mixtures constructed to reflect the specific factor levels of two of these individuals. The empirical reconstruction is consistent with the predicted variation between the two individuals, with the thrombin parameters max rate, max level, clot time parameter and AUC showing the same percentage difference. A good direct fit between empirical and model data is evident for the max rate and clot time parameters but the max level is ∼23% lower in both empirical reconstructions and a difference in AUC is observed. Overall, the correspondence is reasonable and demonstrates the ability of this combined approach of modeling and empirical proteome reconstructions with purified proteins to capture the dynamic consequences of normal range variation in coagulation factor ensembles.

It should be noted that the concept of reasonable correspondence acknowledges the reality of the tension between the variability inherent in the empirical reconstructions and the invariant nature of model parameters. Specifically, synthetic proteome reactions involve 10 purified proteins, natural and recombinant, each reflecting the variability characteristic of any single protein preparation. In contrast, the deterministic mathematical model uses empirically measured rate constants, often averaged from multiple independent studies, and operates as if they, and initial species levels, are known without error [19]. Because of this, model parameters in this study are not adjusted to better fit empirical data from synthetic coagulation proteome experiments.

### Empirical validation of modeling of coagulation proteome variation: responses to anticoagulants

The effects of individual variation in coagulation factor composition on the response to anticoagulants were then assessed. Figure 3 (B-D) shows TGPs from synthetic proteome mixtures, along with corresponding cTGPs, constructed to reflect the specific factor levels of the same two individuals (A and E, see Figure 3A for controls) with either 4 nM, 10 nM or 21 nM rivaroxaban present in the reaction. These data provide examples of the level of fidelity between cTGPs and empirical reconstructions when the kinetic parameters for anticoagulant action are well known. For example, analysis of the cTGPs for individuals A and E indicates that 10 nM rivaroxaban (figure 3C vs figure 3A) prolongs the clot time 2.6 fold, suppresses the maximum level ∼35% and the max rate ∼60%. In the empirical reconstructions the presence of 10 nM rivaroxaban results in an average 2.3 fold prolongation of clot time, a 45% suppression of the max level and a 50% suppression of the max rate. Similar comparisons of cTGPs and empirical reconstructions were successfully performed for each anticoagulant in this study across several concentrations to validate the model descriptions (data not shown). We have previously reported cTGPs and TGPs both constructed with all factors at mean physiologic levels comparing rivaroxaban and Fpx [16].

### Model representations of the alterations in the propagation phase of thrombin generation in individuals subjected to different anticoagulants

The relationship between the variability in the coagulation proteomes of individuals, the mechanisms characterizing different anticoagulants and the relative effectiveness of these anticoagulants in different settings remains unclear. In light of this, modeling based analyses were carried out to systematically compare propagation phase alterations by 5 anticoagulants tested at different concentrations in the same group of individuals (N = 32). Previous reports [16], [20]–[22] and the current study have demonstrated reasonable correspondence between modeled thrombin generation and empirical reconstructions in studies focusing on population differences and anticoagulant efficacy, suggesting that the modeling approach would have some relevance in comparing anticoagulant responses. To carry out this study using the synthetic coagulation proteome would require a minimum of 350 separate experiments and would be unfeasible.

**Figure 4:**
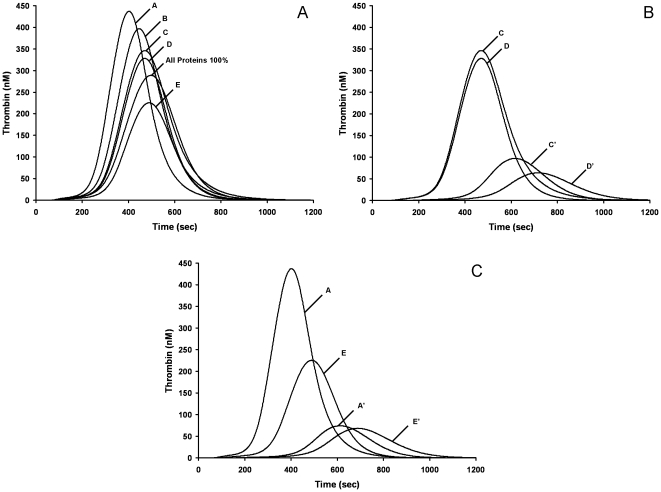
Model representation of hypothetical warfarin anticoagulation of control individuals. cTGPs are presented for individuals before (e.g. A) and after hypothetical warfarin therapy (e.g. A') achieved by setting all VKD proteins at 33% their mean physiologic values with other factors retaining their individual specific values. Panel A: cTGPs of 5 control individuals with an additional cTGP representing an individual with all factors at 100% mean physiologic level. Panel B: 2 individuals with the most disparate thrombin generation profiles prior to anticoagulation. Panel C: 2 individuals with the most similar thrombin generation profiles prior to anticoagulation.

**Figure 5:**
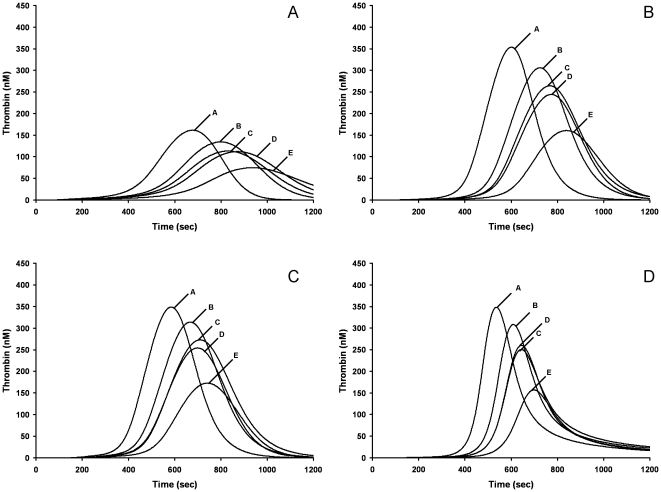
Computational thrombin generation profiles reporting the response of 5 control individuals to anticoagulation. Anticoagulant concentrations are used that yield a group averaged cINR of ∼2.1 for each anticoagulant. Panel A, 1.25 nM UFH. Panel B, 100 nM Fpx. Panel C, 6 nM rivaroxaban. Panel D, 0.5 µM DAPA. Note for these 5 individuals the average sensitivity to Fpx and DAPA was different than the 32 individuals in Table 3.

The consequences of the interaction between variability between individuals in thrombin generation potential (based on plasma coagulation factor composition) and differences in anticoagulant mechanism on propagation phase dynamics are presented for warfarin (Figure 4), two AT dependent agents (UFH, Fpx), rivaroxaban and a reversible thrombin inhibitor (DAPA) (Figure 5).

### Hypothetical warfarin anticoagulation

The average of all four VKD protein levels (Table 1, study 2) in each warfarin-treated group analyzed in Table 1 is ∼30% of its mean physiologic value. Guided by this average distribution, hypothetical warfarin anticoagulation was modeled in the 32 control individuals by setting the VKD protein levels to 25% or 33% that of their mean physiologic value while the concentrations of the other factors were left at their individual specific levels (Table 3). cTGPs for each of the 32 individuals in the larger control group after hypothetical anticoagulation were calculated and expressed relative to the pretreatment thrombin parameter values of that individual to give a percent change in each thrombin parameter. Group averages of these percent change values for each parameter were then generated (Table 3). Thus in these comparisons, unlike those presented in Figure 1, each individual has the same specific level of suppression of VKD proteins. At 33%, the model predicted group average prolongation of clot time was about 2.2 fold (cINR = 2.2) and there was ∼75–80% suppression of all three thrombin parameters. At a 25% VKD protein level, prolongation of clot time for the group was about 2.8 fold (cINR = 2.8) with ∼80–85% suppression of thrombin parameters. The average change in model-derived thrombin parameters reported for the warfarin-treated individuals with average INRs of 2.2 or 2.7 (Table 2: study group 2) were in good accord with the results of hypothetical VKD suppression at 33% and 25% respectively.

**Table 3:** Comparison of anticoagulants at a constant cINR*.

		% Pre-anticoagulation		
Anticoagulant	Fold Prolongation (cINR)	Max Rate	Max Level	AUC
Warfarin 33% VKD†	2.20 (0.13)	19 (3.0)	21 (2.8)	25 (5)
Warfarin 25% VKD†	2.80 (0.15)	12 (1.8)	14 (2.0)	19 (4)
1.25 nM UFH	2.14 (0.11)	19 (1.4)	34 (1.5)	55 (7)
1.75 nM UFH	2.66 (0.14)	13 (1.4)	27 (2.0)	40 (11)
6 nM Riva	2.08 (0.07)	92 (1.3)	71 (1.7)	99 (2)
10 nM Riva	2.60 (0.09)	41 (1.4)	63 (2.0)	90 (8)
0.3 µM DAPA	2.05 (0.05)	114 (3.6)	88 (1.3)	89 (3)
0.6 µM DAPA	2.76 (0.08)	101 (10)	73 (3.0)	72 (5)
125 nM FPX	2.14 (0.05)	87 (2.0)	90 (1.5)	96 (2)
250 nM FPX	2.69 (0.08)	79 (2.8)	84 (2.2)	72 (5)

*A computational analysis of the response of 32 control individuals to the indicated anticoagulants. Thrombin parameters (see Table 2) were extracted and expressed as the ratio of the post- to pre-treatment values. The cINR is the ratio of the thrombin parameter “time to 10 nM” for each anticoagulation concentration. Data are mean (SD), n = 32.

† VKD: Vitamin K dependent proteins in each individual are set to 33 or 25 percent of the mean physiologic levels (other proteins remain at their individual specific levels).

This correspondence between computational representations of actual and hypothetical individuals on warfarin therapy provides a rationale and justification for using the same healthy population to computationally model the efficacy of other anticoagulants and thus compare the responses of the same individuals to different anticoagulant therapies on the basis of their individual coagulation factor ensembles.

Figure 4A presents the cTGPs for five control individuals (study 1) with relatively distinct cTGPs while Figure 4B and 4C show the response of four of the control individuals to hypothetical warfarin anticoagulation. With the two disparate individuals (A and E), equivalent levels of VKD suppression yield cTGPs that differ by less than 10% in the three thrombin parameters. In contrast, in the individuals (C and D) with relatively equivalent cTGPs, an equivalent level of VKD suppression results in a ∼30% difference in all thrombin parameters. Additionally individuals D and E, despite ∼50% differences in all thrombin parameters prior to hypothetical VKD suppression, respond to equivalent levels of VKD protein suppression with ∼superimposable cTGPs. These patterns remain when the same comparisons between individuals at the 25% VKD level are made (not shown). Overall the range of variability in cTGPs among the individuals prior to “treatment” is preserved after hypothetical warfarin anticoagulation but does not directly reflect the relative ranking of thrombin generation intensity prior to “therapy”. The computational analysis makes it clear that normal range variations in plasma factor composition result in unique ensembles of non-VKD dependent proteins that is an important contributor to the anticoagulation phenotype achieved with warfarin and not just the variable levels of VKD proteins that result from actual warfarin therapy (see Table 1). Thus hypothetical warfarin anticoagulation appears to reproduce both the cINR and the specific alterations in thrombin parameters characterizing actual warfarin-treated populations as well as the unpredictability (variability) of the outcome [15].

### Anticoagulation by mechanistically distinct inhibitors

Computational TGPs reporting the “responses” of the same 32 control individuals to anticoagulation by UFH, Fpx, rivaroxaban, and DAPA were generated for a number of concentrations of each anticoagulant. The concentrations of each anticoagulant which gave a group averaged cINR of ∼2.1 and of ∼2.7 were selected for comparison to the cTGPs for the same individuals undergoing hypothetical warfarin therapy. This allowed the comparison of propagation phase parameters at the same clot time prolongation value (i.e. the same INR). These data are summarized in Table 3.

### UFH

Active heparin molecules, constituting 30 to 40% of UFH preparations, bind to and conformationally activate the stoichiometric inhibitor AT, increasing its reactivity with thrombin (10^3^ fold) [23], fXa (10^4^ fold) [24], [25], fIXa (10^6^ fold) [26] and Tf-fVIIa (20 fold) [27]. A large percentage of UFH molecules appear to bind to plasma proteins and various blood cells [28] (Table 4).

**Table 4:** Properties of Anticoagulants.

	Chemical Expression	Clinical Dose Range(Prophylaxis)
UFH	AT+UFH 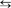 AT-UFH	0.1 – 0.4 U/mL^a^
	Xa+AT-UFH→Xa-AT+UFH	
	IXa+AT-UFH→IXa-AT+UFH	
	mIIa+AT-UFH→mIIa-AT+UFH	
	TF-VIIa+AT-UFH→TF-VIIa-AT+UFH	
	Va-Xa+AT-UFH→Va-Xa-AT+UFH	
	IIa+AT-UFH→IIa-AT+UFH	
Fpx	AT+F 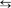 AT-F	0.12 – 0.4 µM^b^
	Xa+AT-F→Xa-AT+F	
	IXa+AT-F→IXa-AT+F	
	mIIa+AT-F→mIIa-AT+F	
	TF-VIIa+AT-F→TF-VIIa-AT+F	
	Va-Xa+AT-F→Va-Xa-AT+F	
	IIa+AT-F→IIa-AT+F	
Rivaroxaban	Xa+R 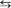 Xa-R	0.02 – 0.31 µM^c^
	Va-Xa+R 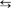 Va-Xa-R	
DAPA	IIa+DAPA 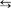 IIa-DAPA	0.6 – 1.8 µM^d^
	mIIa+DAPA 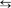 mIIa-DAPA	0.08 – 0.39 µM^e^
	mIIa-DAPA+Xa-Va→IIa-DAPA+Xa-Va	

a 1U ≡ 170 nM functional heparin molecules; in blood, >90% of UFH may be unavailable for binding with AT [28], [45].

b Fpx binds almost exclusively to AT in blood [34], [46].

c C_trough_ – C_max_ (10 mg once daily): 5 – 8% free in plasma [47], [48].

d Dose ranges for Argatroban, a structurally related reversible thrombin inhibitor in clinical use (K_D_ = 20 nM) [49].

e C_trough_ – C_max_ for Dabigatran (220 mg once daily), a reversible thrombin inhibitor (K_D_ = 50 pM) [50], [51].

Figure 5A presents representative cTGPs in the presence of 1.25 nM UFH (0.01 units/mL) for 5 selected individuals A to E, with the group (N = 32) averaged changes in thrombin parameters presented in Table 3. The group averaged cINR for these cTGPs was 2.1±0.1, comparable to that achieved with these individuals at a hypothetical warfarin induced VKD level of 33% (cINR = 2.2±0.1). When propagation phase features of UFH anticoagulation were evaluated, this concentration of UFH yielded reductions in the thrombin parameter max rate similar to those observed with the hypothetical warfarin group and to those observed with the actual warfarin group (study group 2, INR 2–2.4, Table 2). However the suppression of the parameters max level and total thrombin by UFH was not as robust as in both hypothetical and actual warfarin groups. At a concentration of UFH that yielded a cINR of 2.7, approximately equivalent to that observed with hypothetical warfarin induced VKD level of 25%, the same pattern was observed, with UFH anticoagulation showing distinct differences from warfarin anticoagulation in the same two propagation phase parameters. In addition, unlike hypothetical warfarin therapy, the response to UFH is relatively uniform between individuals, with the pretreatment order of relative intensity of thrombin generation among the 5 individuals (see Figure 4A) preserved with UFH anticoagulation. For example suppression of the parameter max level in individual A is 35%, while in individual E it is 34%. This contrasts with the response to hypothetical warfarin coagulation (Figure 3A) where max level is suppressed ∼85% in individual A and ∼33% in individual E.

### Fpx

Fpx is a synthetic pentasaccharide that reversibly binds with high affinity (K_D_ = 36 nM [29]) to AT and increases its rate of inhibition of fXa [23] and fIXa [26] ∼100 fold (Table 4). Figure 5B presents cTGPs for same individuals A to E in the presence of 125 nM Fpx: the group averaged cINR is 2.1±0.1 (N = 32), similar to that characterizing a hypothetical warfarin induced VKD level of 33%, UFH at 1.25 nM and the actual warfarin treated group (Table 2, study group 2:INR 2.0–2.4). As can be seen by both visual inspection (compare to Figures 4, 5A) and in thrombin parameter data (Tables 2, 3), Fpx anticoagulation at equivalent cINR displays little suppression of thrombin parameters max rate and level and almost no suppression of total thrombin. The same relative lack of suppression of propagation phase parameters is observed when the group averaged thrombin parameters at a cINR of 2.7 are analyzed. Similar to UFH anticoagulation, the response to Fpx is relatively uniform between individuals. Thus features of Fpx anticoagulation differ substantially from those of UFH and warfarin when compared at equivalent cINRs.

### Rivaroxaban

Rivaroxaban is a small molecule, reversible inhibitor that selectively binds to the active site of fXa and fXa in the prothrombinase complex [30]. Figure 5C presents cTGPs in the presence of 6 nM rivaroxaban for individuals A to E: the group averaged cINR is 2.1±0.1 (N = 32). The overall quality of this anticoagulation is quite similar to that achieved by 125 nM Fpx, with the two anticoagulants showing almost equivalent suppression of the thrombin parameters max level and max rate and a similar lack of effect on total thrombin generated. Some differences emerge between rivaroxaban and Fpx anticoagulation when their thrombin parameters accompanying a cINR of 2.7 are compared with rivaroxaban, having more pronounced suppression of the parameters max rate and level. Like UFH and Fpx anticoagulation, the response to rivaroxaban is uniform between individuals, consistent with the results of the empirical reconstructions comparing anticoagulation by rivaroxaban of individuals A and E (Figure 3, B-C).

### DAPA

DAPA is a reversible, active site directed inhibitor of thrombin and meizothrombin (mIIa), with a K_D_ of 20–40 nM [31], [32]. The mIIa = DAPA intermediate [31] is processed to IIa = DAPA by prothrombinase. Similar to Argatroban [32], a structurally related thrombin inhibitor in clinical use, the k_off_ for the DAPA thrombin complex is in the range of 0.04 to 0.07 s^−1^ (in house) (Table 4).

Figure 5D presents cTGPs in the presence of 0.3 µM DAPA. Examination of DAPA anticoagulation of individuals A to E shows it lacks the effectiveness of either warfarin or UFH in suppressing propagation phase parameters when compared at either a group averaged cINR of 2.1 or 2.7 (Table 3). Relating its efficacy to those of rivaroxaban and Fpx is less straightforward. At a cINR of either 2.1 or 2.7, DAPA anticoagulation appears similar to Fpx with respect to its effects on thrombin parameters max level and total thrombin but differs in its striking ineffectiveness in suppressing the parameter max rate. The level of thrombin parameter suppression by DAPA is relatively uniform among the five individuals. In general the features of DAPA anticoagulation distinguish it from the other anticoagulants in this study.

### Summary of anticoagulant effects on the propagation phase

The average thrombin generation phenotype associated with warfarin anticoagulation reflects a more robust suppression of all 4 thrombin parameters than any of the other anticoagulants. The relative order of suppression displayed at the same cINR was: warfarin > UFH > DAPA ≥ Fpx, rivaroxaban.

## Discussion

In this study, we first compare model representations (*i.e.* cTGPs) based on plasma coagulation factor composition data from actual individuals successfully treated with warfarin to cTGPs characterizing matching control populations. A metric—the computational INR (cINR)—is defined and shown to correlate well with the actual INR values of these individuals, thus providing validation of the computational approach using the one clinical measure available for the warfarin-treated individuals. When analyzed on an individual by individual basis differences between clinical INR and cINR values derive primarily from variations in TFPI levels, to which the PT assay is insensitive.

The dependence of the discrepancy between the cINR and plasma INR on the magnitude of the difference between an individual's TFPI level and the mean physiologic TFPI level suggests a testable hypothesis: individuals with large differences between INR and cINR are not at the expected level of anticoagulation and should be more prone to adverse events. Negative deviations in the (cINR-INR)/INR metric, associated with low TFPI levels, suggest under-anticoagulation and thus a risk of thrombotic events while large positive deviations would indicate a relatively increased bleeding risk. Consistent with this implication of the computational analysis, the 3 individuals that had thrombotic complications did display cINRs <2, strong negative deviations in the (cINR-INR)/INR metric and low TFPI levels. However thrombotic complications were not reported for 5 other individuals with similar parameters. A larger study would be required to test the predictive capacity of this approach.

A central finding of these analyses is that none of these other anticoagulants recapitulate all features of warfarin anticoagulation when defined in terms of the pattern of propagation phase thrombin formation. This conclusion is driven by the mechanistic descriptions of each inhibitors interactions and is not limited to the specific binding parameters and concentration ranges used in the analyses of the examined inhibitors. Thus modeling results apply generally to the entire classes of reversible thrombin inhibitors, reversible fXa inhibitors, and low molecular heparins with mechanisms of action similar to Fpx. For example, the direct thrombin inhibitor dabigatran (Table 4) reproduces the pattern of propagation phase parameter suppression observed with DAPA when analysed at a cINR of 2.1 (data not shown).

The current analyses show that propagation phase parameters (Table 3) are less affected by Fpx, rivaroxaban and DAPA than warfarin and UFH when compared at concentrations that yield equivalent cINR. Empirical reconstructions presented in this study and previously [16] confirm the model predictions of relatively robust propagation phase parameters for rivaroxaban and Fpx relative to warfarin. Empirical reconstructions (not shown) of DAPA and UFH anticoagulation are also consistent with model representations. Similar results, *i.e.* relatively robust propagation phase thrombin parameters at anticoagulant doses that prolong the lag phase 2 to 3 fold, have been reported for dabigatran [12], Fpx [13] and rivaroxaban [33] when assessed by plasma-based thrombin generation assays.

In our previous study [16] comparing Fpx and rivaroxaban efficacies in computational, synthetic coagulation proteome and whole blood models, we observed that Fpx efficacy was relatively consistent between the 3 models and was in line with the therapeutic usage range of Fpx. However, with rivaroxaban 10 to 20 times higher concentrations were required in whole blood studies than predicted by computational and matching proteome reconstructions, with these higher levels being in line with plasma levels achieved during standard clinical use of rivaroxaban. We hypothesized that these differences reflected the different functional distributions of rivaroxaban and Fpx in the blood: Fpx is almost exclusively bound to AT in human plasma [34] and therefore fully available to participate in anticoagulation even in whole blood while ∼90 to 95% of rivaroxaban is bound to plasma proteins [2], with the unbound fraction representing the pharmacologically available fraction. Similarly, but in contrast to Fpx, UFH exhibits strong binding to plasma proteins other than AT as well as cellular elements [28]. Studies in our laboratory (data not shown) testing UFH efficacy in whole blood show that to achieve a ∼2 fold prolongation in clot time, a UFH concentration (0.05 to 0.1 U/ml) 5 to 10 times higher than in proteome and computational models is required. Collectively, these data suggest that the computational assessments of inhibitor efficacy may represent the actual levels of anticoagulant in blood that are dynamically available to suppress coagulation *in vivo.*

The significance of anticoagulant effects on clot time and propagation phase thrombin generation needs to be viewed in the context of 2 different environments: one is intravascular with blood flowing over an emerging procoagulant lesion in a diseased region of a blood vessel; and the other is the extravascular space to which blood is transferred following perforating trauma to a blood vessel [16]. In the first setting anticoagulation seeks to supplement the natural antithrombotic properties of a vessel—the mechanical and dilutional effects of flow and the antithrombogenic effects (thrombomodulin, TFPI) contributed by neighboring endothelial cells—by further reducing the inherent coagulability of the blood. The desired result is to arrest the Tf-initiated process in the initiation phase so that thrombus formation is prevented. Thus *in vitro* tests focusing on clot time prolongation appear to be a reasonable approach on theoretical grounds for calibrating anticoagulant doses for this setting. Conversely tests that focus on differences in propagation phase dynamics between inhibitors may not be relevant in this context.

The second environment, the extravascular, is one both relatively rich in Tf and the proteins that support platelet adhesion while somewhat physically isolated from the antithrombotic forces of the vascular environment. Here it is desirable in the context of long-term anticoagulation that the anticoagulated blood supports formation of an adequate clot in response to unexpected perforating trauma to the blood vessel in an anticoagulated individual. Thus here the criterion of clot time prolongation does not appear relevant to comparing the relative suitability of anticoagulants. However, to the extent that the quality of post-clot thrombin generation is important to fibrin structure, barrier maintenance and the wound healing processes [7], [8], anticoagulant effects on the propagation phase of thrombin generation may represent an important discriminator for understanding the relative levels of unwanted bleeding episodes associated with different anticoagulants. The data from this study suggests that thrombin and factor Xa directed inhibitors may have mechanistic-based advantages over the more broadly based inhibitory effects of warfarin and UFH therapies in this context since at doses that give equivalent prolongation of clot times (cINR values), propagation phase thrombin generation, once it begins, is more robust.

Successful long-term anticoagulant therapy occurs when a balance is achieved between suppression of catastrophic procoagulant responses at specific compromised site(s) within the vasculature and maintenance of a reasonable level of repair response to perforating trauma to the circulatory system. For any anticoagulant, the ability to balance these two opposing goals at a single dose theoretically derives in part from the differences between the two settings in the size of the procoagulant stimulus. For example, Tf expression contained within atherosclerotic plaques has been reported as ∼6 molecules/µm^2^ [11] while a Tf surface density of ∼60 molecules/µm^2^ has been reported for human fibroblasts [35]. In the empirical studies presented here—5 pM surface available Tf and 2 µM PCPS vesicles—there are 3 to 4 Tf molecules/µm^2^ of solution exposed phospholipid, comparable to the estimates for the atheroma but not the extravascular milieu.

In order to provide a quantitative estimate of the impact of differential expression of Tf on propagation phase parameters for different anticoagulants, a preliminary computational analysis (Table 5) was undertaken to establish what Tf concentration was necessary to normalize the clot time in the presence of each anticoagulant when the anticoagulant was present at a concentration designed to give a cINR of ∼2.1 at 5 pM Tf. The propagation phase parameters of thrombin generation could then be compared to those characterizing the 5 pM Tf stimulus in the absence of anticoagulants to see which anticoagulants achieved “normal” thrombin generation in the extravascular compartment. In general increased Tf concentrations of only 2 to 4 fold were needed to achieve the standard clot time in the absence of anticoagulants, independent of the mechanism of the anticoagulant. With rivaroxaban, Fpx and DAPA this resulted in propagation phase parameters as robust as or more so than that of the control. However, in the case of warfarin and UFH anticoagulation all propagation phase parameters remained suppressed by ∼2 fold or more.

**Table 5:** Anticoagulant efficacy and Tf concentration*.

Anticoagulant	TF Conc.(pM)	Clot Time(s)	Max Level(nM)	Max Rate(nM/s)	AUC(nM*s)
Control	5 pM	173	436	3.4	93553
33% VKD†	5 pM	371	97	0.7	25667
33% VKD†	17.5 pM	177	151	1.6	25175
1.25 nM UFH	5 pM	322	161	0.7	53309
1.25 nM UFH	9 pM	176	205	1.3	48051
6 nM Riva	5 pM	358	323	1.8	94673
6 nM Riva	17.5 pM	174	484	4.3	93250
0.6 µM DAPA	5 pM	379	327	3.8	75670
0.6 µM DAPA	17.5 pM	176	450	8.3	79422
125 nM FPX	5 pM	360	334	1.9	91273
125 nM FPX	12.5 pM	171	477	4.2	89708

*Thrombin parameters were extracted from cTGPs at the indicated anticoagulant and Tf concentrations.

† VKD: Vitamin K dependent proteins for individual A are set to 33 percent of the person's physiologic levels (other proteins remain at their individual specific levels).

In summary, our results indicate that computational methods focusing on thrombin generation can discriminate between different anticoagulants compared at equivalent cINR and between different individuals on the same anticoagulation regimen, supporting the idea that this approach can contribute to the management of anticoagulant therapy. The question with the use of anticoagulants, or more generally at all therapies directed at redressing blood clotting disorders, is how much you need to know about each individual's coagulation system to improve outcomes. Empirical thrombin generation assays require only citrate plasma samples and samples of potential anticoagulants to provide an immediate readout of thrombin generation profiles and dose response data. However, as with the PT assay, those readouts, whether characterized as unusual or typical, are opaque as to the origins of their features and as to why one individual appears the same or different from another. For example, a recent study has shown significant inter-individual variability in the response to different anticoagulants when assessed by a thrombogram methodology [14]. The modeling based approach requires coagulation factor analyses of each individual's citrate plasma sample, but yields a representation of an individual's coagulation state that is easy to dissect, based on dynamics reflecting proteins at their physiologic concentrations and native conformations and well established mechanisms for the anticoagulants. It is amenable to rapid screening of the efficacy of available anticoagulants at a variety of concentrations or possible combinations. Advances in measurement technology to reduce the cost of coagulation factor composition analysis should make this approach more feasible and enable pre and post treatment assessments that should further individualize anticoagulant therapy.

## Materials and Methods

### Ethics

Population study 1 (dating to 2002) was approved by the Institutional Review Board Committee on Human Research in the Medical Sciences at the University of Vermont. All participants gave informed written consent.

Population study 2 was approved by the Jagiellonian University Ethical Committee. All participants gave informed written consent.

### Materials

Human fV, fVII, fVIIa, fIX, fX, antithrombin (AT) and prothrombin were either obtained from Haematologic Technologies (Essex Junction, VT) or purified from fresh human plasma using modifications of the methods of Bajaj *et al.* (fVII, fX, fIX and prothombin) [36], Katzmann *et al.* (fV) [37] and Griffith *et al.* (antithrombin) [38] or received as a gift (recombinant human fVIIa) from Dr. Ula Hedner (Novo Nordisk, Denmark). Recombinant fVIII, tissue factor pathway inhibitor (TFPI) and recombinant tissue factor (Tf) (residues 1–263) were purchased from American Diagnostica, Inc (Greenwich, CT) or received as gifts: recombinant fVIII and recombinant Tf (residues 1–243) from Drs. Shu Len Liu and Roger Lundblad (Hyland division, Baxter Healthcare Corp, Duarte, CA) and recombinant full-length TFPI from Dr. S. Hardy (Chiron Corp, Emeryville, CA). 1,2-Dioleolyl-*sn*-Glycero-3-Phospho-L-Serine (PS) and 1,2-Dioleoyl-*sn*-Glycero-3-Phosphocholine (PC) were purchased from Avanti Polar Lipids, Inc (Alabaster, AL). Phospholipid vesicles (PCPS) composed of 75% PC and 25% PS were prepared as described [39]. Spectrozyme TH was purchased from American Diagnostica, Inc (Greenwich, CT). The preparation of the Tf/lipid reagent was performed as described [40]. Rivaroxaban (Xarelto®) was provided by Bayer HealthCare AG, Leverkusen, Germany.

### Populations

Study 1: Individuals have been described previously [15]: 9 male patients (68±12 years) on stable warfarin anticoagulation (0.16 to 10 years); and 5 apparently healthy male subjects (51±7 years). Study 2: 47 patients (19 females, 28 males; age: 25-75 years) on stable anticoagulation (mean time of 4 months) with warfarin (2≤INR≤3). Indications for vitamin K antagonist administration were atrial fibrillation (N = 20), venous thromboembolism (N = 20) or aortic prosthetic valve implantation (N = 7). The exclusion criteria were recent (preceding 6 months) thromboembolic event, acute infection, liver injury, renal insufficiency, autoimmune disorders or known cancer. Three individuals had a thrombotic event subsequent to the blood draw for compositional analysis. Thirty-two apparently healthy individuals recruited from hospital and university staff (Jagiellonian University Medical College, Krakow, Poland) served as controls.

### Blood Collection and Coagulation Protein Analyses

Fasting blood was drawn into 0.1 volume of 3.2% trisodium citrate from an antecubital vein with minimal stasis. Citrated blood samples were centrifuged within 15 minutes of collection and stored in aliquots at –80 °C until further use. Factors II, V, VII, VIII, IX, X were measured by one-stage clotting assays (Dade Behring, Liederbach, Germany) using factor-deficient plasmas. AT activity was measured using a Berichrom chromogenic assay (Dade Behring). Free TFP) was determined using an ELISA (Diagnostica Stago, Asnieres, France).

### Computational Model

Computational simulations of Tf-initiated thrombin generation profiles (cTGPs) were produced as described previously, with rate constants reflecting measurements made at saturating phospholipid for each phospholipid dependent process [16], [41].

### Incorporating pharmacologic agents

Anticoagulants were modeled by adding the appropriate sets of equations (see Table 4) describing their activities to the existing framework of differential equations. The rate constants employed were as follows: UFH reaction with AT [26], [29]; UFH-AT reactions with fXa [24], [42], [43], fIXa [26], meizothrombin [44], Tf-VIIa [27], fXa-fVa [43], and thrombin [44]; and Dansylarginine-N-[3-ethyl-1,5-pentanediyl]amide (DAPA) reaction with thrombin and meizothrombin [31]. Modeling of Fpx and rivaroxaban interactions with their targets was conducted as described previously [16].

### Modeling warfarin-treated individuals

A computationally derived thrombin generation profile (cTGP) based on each warfarin-treated or control individual's coagulation factor composition was generated. Thrombin parameters (max rate, max level, total thrombin (area under the curve, AUC) and time to 10 nM total thrombin, the computational equivalent of clot time in our empirical models) were extracted as described previously [18] from each cTGP. Thrombin parameters from each warfarin-treated individual (for example study 2: INR 2.0–2.4: N = 25) were then ratioed to the corresponding parameters from every member of the relevant control group (study 2, N = 32), thus generating a characterization of each warfarin-treated individual's state of anticoagulation relative to the variation in thrombin generation in a normal population. The means and standard deviations for the four thrombin parameters, representing the entire ensemble of 800 values for each parameter were then calculated.

With this approach, the time to 10 nM total thrombin parameter allows a comparison of model predicted clot times between each anticoagulated individual and all control individuals, yielding a ratio value (the computational or cINR) analogous to the ratio between a patient's prothrombin time and that of a standard plasma sample (INR).

### Analyzing differences between the INR and cINR of warfarin-treated individuals

The coagulation factor dependence of differences between clinical INR and cINR values in each warfarin-treated individual was investigated by establishing a metric defined as the (cINR-INR)/INR, which quantified the relative disparity between model and PT assay derived values. Each individual specific difference was plotted against the corresponding level of a specific factor. A series of plots testing all 8 factors was constructed and linear regression analyses performed.

### Hypothetical anticoagulation of 32 control individuals

Thrombin parameters were extracted as described previously [18] from each cTGP representing a given individual from the control population on a specific dose of an anticoagulant. In the case of hypothetical warfarin anticoagulation, the concentrations of the vitamin K dependent (VKD) proteins (fII, fVII/VIIa, fIX and fX) were set to 25% or 33% their mean physiologic values while the other factor levels remained at their individual specific concentrations. Each thrombin parameter was then expressed relative to the pretreatment value of that parameter for that individual, yielding either a cINR or a percent change for other parameters at a specific dose of anticoagulant. Group averages for a given concentration of anticoagulant were then calculated for each parameter using the ratio values.

### Synthetic Coagulation Proteome

The procedure used has been described previously [6], [16]. Relipidated Tf reagent at 5 pM final concentration was added to a mixture of fII, fV, fVII, fVIIa, fVIII, fIX, fX, TFPI, and AT in 20 mM HEPES/150 mM NaCl with 2 mM CaCl_2_ containing 2 µM PCPS. Concentrations of factors and inhibitors were set to those characterizing specific individuals. Pharmacologic agents were incorporated into the reaction mixture prior to addition of the Tf reagent.
